# Comparison of the application value of bipolar electrocoagulation and ultrasonic scalpel in robot-assisted thyroid carcinoma surgery: a single-center study based on perioperative indicator and short-term prognosis

**DOI:** 10.3389/fsurg.2026.1764805

**Published:** 2026-02-12

**Authors:** Yanxu Dong, Liqun Huo, Lu Wang, Jun Gu, Bing Xie, Xiaofan Xu

**Affiliations:** 1 Research Institute of General Surgery, Affiliated Jinling Hospital, Medical School of Nanjing University, Nanjing, China; 2 Department of General Surgery, Midong District People' Hospital of Urumqi, Urumqi, Xinjiang Uygur Autonomous Region, China

**Keywords:** bipolar electrocoagulation technique, Da Vinci robotic surgery, postoperative complications, short-term prognosis, thyroid carcinoma

## Abstract

**Objective:**

To study the effectiveness of bipolar electrocoagulation cutting technology and traditional ultrasonic scalpel cutting technology in Da Vinci robot-assisted thyroid carcinoma surgery.

**Methods:**

We retrospectively reviewed 154 patients with thyroid carcinoma who had undergone ultrasonic scalper (US, *n* = 81) or Maryland bipolar electrocoagulation (MB, *n* = 73) cutting in Da Vinci robotic surgery. The operation time, complications, efficacy and other clinical indicators were compared between the two groups.

**Results:**

The operation time of the MB group was significantly reduced (*p* < 0.05), and the drainage volume was significantly higher than that of the US group (*p* < 0.05). The postoperative PTH and serum calcium levels of total thyroidectomy in the MB group were higher than those in the US group (*p* < 0.05), which were (US: 2.07 ± 1.51, MB: 2.42 ± 1.46) and (US: 2.15 ± 0.14, MB: 2.20 ± 0.13).

**Conclusion:**

Bipolar coagulation cutting technology is more precise. Its application in robotic thyroid surgery can significantly shorten the operation time and protect parathyroid function, which has better clinical application prospects.

## 1 Introduction

Thyroid carcinoma is one of the most common endocrine malignancies (1). It is more common in people aged 30–50 years, and is approximately four times more common in women than in men (2). At present, surgery is the main treatment for thyroid tumors (3). Postoperative scars have become a common focus with improvements in the population's demand for treatment quality. To avoid scarring in the anterior neck from thyroid surgery, surgical specialists have developed various minimally invasive remote access pathways (4). From traditional open surgery to the introduction of Da Vinci Surgical Robot, thyroid surgery has become more refined with the high definition of video system and the refinement of operating instruments. Robotic thyroid surgery methods have been demonstrated in large-scale clinical studies of surgical safety, complications, and tumor safety (5). However, the recurrent laryngeal nerve(RLN) and parathyroid gland may be damaged by thermal injury of energy devices during surgery, which may affect postoperative recovery (6). Therefore, the cornerstones of thyroid surgery are capsular dissection of the thyroid gland as well as the skilled use of energy instruments. The thyroid gland is covered with a complete capsule, and the outer layer is a loose pseudo-capsule composed of the deep cervical fascia, which is closely connected to the vascular sheath of the neck. The inner layer is a fibrous capsule that adheres closely to the surface of the thyroid gland and forms an authentic capsule. The true capsule extends further to emit small septa into the thyroid parenchymal result in dividing the glandular tissue into multiple small lobulated structures (7), that contributes to the fixation of the thyroid gland and eventually merges with the lateral ligament and isthmus of the thyroid gland. The lateral ligament connects the lateral lobe of the thyroid to the tracheal ring, whereas the isthmus connects the two lateral lobes of the thyroid to form a complete structure. The lateral ligaments and the isthmus form the suspensory ligaments that hold the thyroid gland to the trachea and larynx.

The development of capsular anatomy techniques is attributed to the advancements in endoscopy and the use of instruments, enabling surgeons to clearly observe more microscopic anatomical structures than those of blood vessels, and to perform precise anatomical dissections in specific fascial planes to achieve the ligation and clearance of blood vessels and lymphatic vessels. As early as 1907, Halsted and Evans proposed to perform “super microscopic dissection” in the ventral capsule area close to the true capsule of the gland without ligation of the main trunk of the inferior thyroid artery to fully preserve the parathyroid gland and its blood supply (8). Using ultrasonic scalpels for layer-by-layer dissection, from the fascia between the sternocleidomastoid muscle and the internal jugular vein, to the superficial and middle layers of the deep fascia between the common carotid artery middle segment and the thyroid gland, to the fusion fascia between the thyroid gland and the central area tissues, and finally, along the thyroid inferior artery, from the posterior side of the carotid sheath, to the anterior space of the anterior space of the anterior scalene fascia. On the surface of the thyroid capsule, parathyroid glands were searched along the extension of the inferior thyroid artery branch. The path could be planned according to the anatomical relationship between the inferior thyroid artery and parathyroid gland after finding the parathyroid gland, and the graded blood supply vessels from the inferior thyroid artery were retained (9). Ultrasonic scalpel (US) is an important anatomical tool in both minimally invasive endoscopic surgery and robotic surgery (10). However, US in Da Vinci robotic surgical system has limited flexibility due to the lack of internal joints. And it has a thick cutting surface and a large range of thermal radiation (11). Bipolar electrocoagulation is used to coagulate and cut tissue, not only because its electrocoagulation hemostasis effect is superior to that of unipolar electrocoagulation equipment, but also the use cost was lower than that of ultrasonic scalpel (12). In view of the difference between the two energy instruments, “Maryland Bipolar Forceps” has been used to dissect free thyroid since January 2024 on the premise of determining the safety of the operation. To determine whether this improvement measure will improve the surgical effect, a retrospective study method was used to compare clinical efficacy, safety and postoperative recovery between the group of patients who used ultrasonic knife before January 2024 and bipolar electric coagulation forceps after January 2024 for robotic thyroid cancer surgery. Better surgical results and fewer surgical complications were expected.

## 2 Materials and methods

### 2.1 Patients

**Figure 1:**
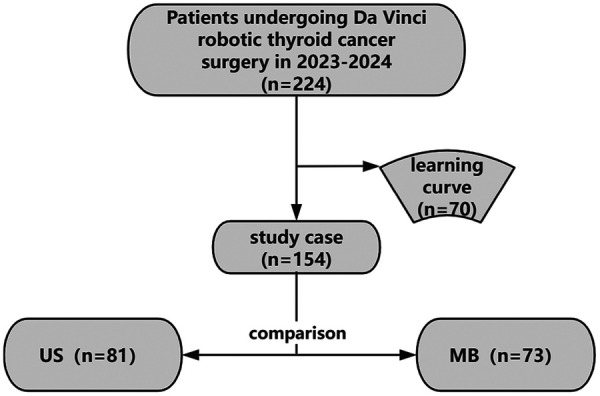
Patients grouping. 224 patients with thyroid cancer underwent Da Vinci robotic thyroid carcinoma surgery from March 2023 to December 2024,of which the first 70 cases were excluded due to the learning curve. The cohort was divided into an ultrasonic scalpel group (US: *n* = 81) and a Maryland bipolar coagulation group (MB: *n* = 73).

A total of 224 patients with thyroid carcinoma had undergone Da Vinci robotic thyroid cancer surgery since Jinling Hospital Affiliated to Medical School of Nanjing University began to carry out from March 2023 to December 2024. The patients were divided into the US group and the MB group shown as Figure 1. All cases were performed by the same surgical team. Inclusion criteria: (1) pathological characteristics of thyroid carcinoma confirmed by cytology and pathology; (2) tumor size ≤2 cm and no extrathyroidal invasion; (3) no fused, fixed or cystic lymph nodes in the neck, and no lymph node metastasis in domain VII. (4) patients with no other tumors; (5) Meeting the surgical indications; (6) Patients had informed consent and strong desire for cosmetic treatment. Exclusion criteria: (1) previous history of neck surgery or radiotherapy; (2) deformities of the neck or chest; (3) had other metabolic diseases; (4) pregnant or lactating; (5) had cardiopulmonary dysfunction or severe coagulation dysfunction and could not tolerate general anesthesia and surgery. The ethics committee of our hospital has reviewed and approved this study (Ethics number: 2023DZYNKT-001).

### 2.2 Surgery

All patients were treated with nanocarbon and indocyanine which was named green double tracer technique. The patients were injected with carbon nanoparticles into the thyroid tissue under ultrasound guidance 12–24 h before surgery. The cervical lymph nodes were visualized and the parathyroid glands were “negatively visualized” during the operation (13). Preoperative indocyanine green allergy testing was also performed to allow intraoperative real-time “positive visualization” of the parathyroid gland (14). During the operation, the RLN was detected in real time via an endotracheal tube with neuromonitoring. The operation table was adjusted to raise the upper part by 15° and the head was lowered by 15° after anesthesia. If necessary, a silicone shoulder pad was used to maintain the posterior supine position, and the head ring and neck pad were fixed to protect the head and neck. The puncture tunnel and surgical area were subsequently marked by marker lines. After disinfection, we prepared 80–100 mL of swelling solution (500 mL of normal saline and 1 mg of epinephrine) for subcutaneous injection into the anterior part of the stem of sternum and the pre-marked puncture path. A 5–12 mm incision was made at the medial and upper sides of the bilateral areola and the anterior axillary fold of the bilateral axilla. The subcutaneous tunnels were bluntly separated by a subcutaneous separating rod to the suprasternal fossa. The puncture apparatus was connected to each of the four robotic arms. We connected the robotic arm at the right areola to the lens and connected the other three arms to the electric hook/US, grasping forceps, and MB. The MB was connected to the recurrent laryngeal nerve electrode for real-time intraoperative monitoring signal. The cervical white line was incised with an electric hook/US, and the sternothyroid muscle and sternohyoid muscle were retracted with grasping forceps and separating forceps to expose the thyroid isthmus and the affected gland lobe. When a suspected parathyroid gland was found during the operation, we injected indocyanine green intravenously to distinguish (15).

**Figure 2:**
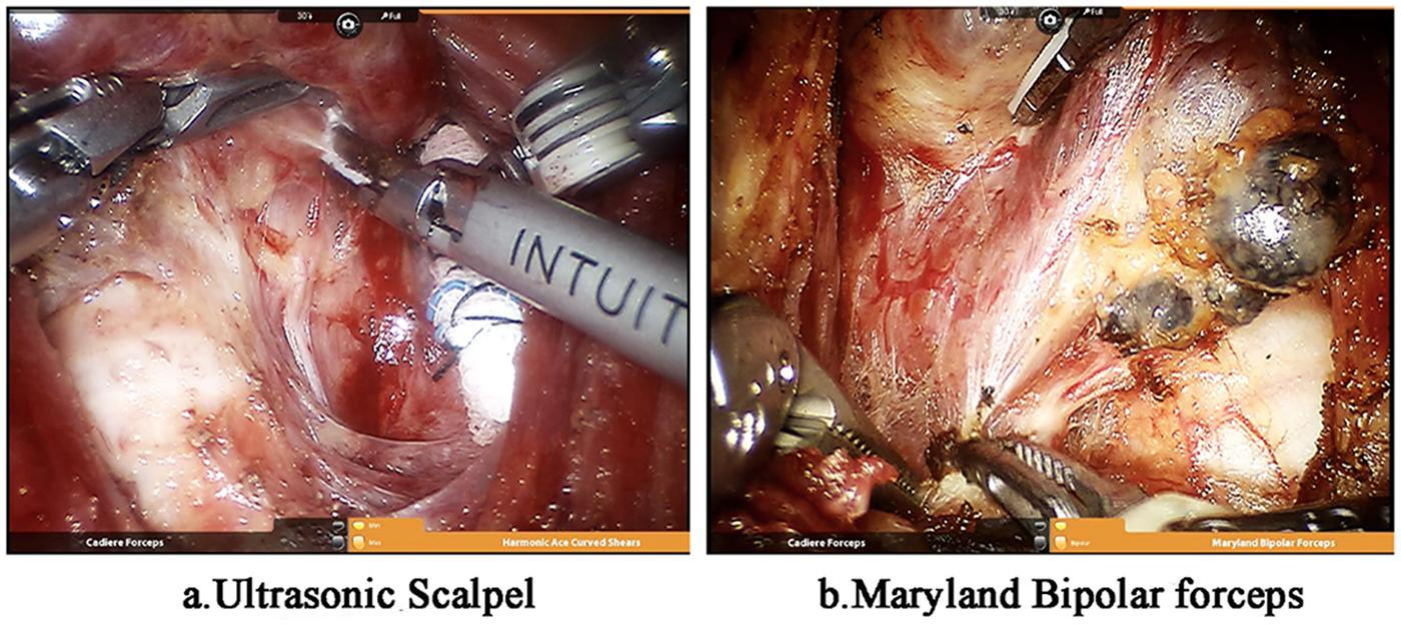
Da vinci thyroidectomy **(a)** Ultrasound scalpel was used for thyroid capsule coagulation and dissection; **(b)** Maryland bipolar electrocoagulation forceps was used for thyroid capsule coagulation and dissection.

In the US group, the thyroid gland was dissected with an ultrasonic scalpel (Figure 2a). The thyroid isthmus was dissociated along the trachea and cut off. The upper blood vessels were cut off upward, and the lower blood vessels and the middle vein were cut off downward along the surface of the thyroid gland. In the MB group, the thyroid capsule was dissected and separated with Maryland bipolar electrocoagulation (Figure 2b). The thyroid isthmus was dissociated along the trachea, the superior blood vessels were cut upward, the inferior blood vessels and the middle vein were separated downward along the thyroid capsule, the visceral mesentery of the thyroid was dissected along the mesentery space, and all the mesentery structures including thyroid suspensory ligament and lateral thyroid ligament were separated. The lobe and isthmus of the affected side were cut until the place where the recurrent laryngeal nerve entered the larynx. For some patients who underwent total resection, the contralateral lobe was removed in the same way.

When we dissected the central lymph node, the trachea was pulled to the opposite side with grasp forceps, and the black-stained lymph nodes in the central region were separated from the lymphatic adipose tissue by US or MB. The scope of dissection was down to the point where the inname artery crossed the trachea, up to the level of the hyoid bone, lateral to the common carotid artery, and medial to the trachea. Then indocyanine green was injected again to determine the blood supply of the parathyroid gland. If the blood supply was poor, the parathyroid homogenate was injected into the deltoid muscle (16). The recurrent laryngeal nerve probe was used to detect and protect the recurrent laryngeal nerve during the whole operation (17).

### 2.3 Observation indicators

(1) The operation time, length of stay (LOS), degree of bleeding, postoperative drainage volume, and incidence of postoperative complications were compared between the two groups. (2) Five milliliters of fasting cubital venous blood was collected before the operation and 1 day after operation to determine the serum PTH level and the patient's serum calcium level. (3) RLN and cervical parasympathetic nerves injury.

### 2.4 Statistical analysis

SPSS V21.0 (IBM Corporation, Armonl, NY, USA) was used to analyze the data and GraphPad Prism 8.0 was used to generate graphs. The measurement data were expressed as the mean ± standard deviation ($\bar{x}\pm s$) and were analyzed via independent-samples *t*-test. Count data were expressed as the number of cases (n) and analyzed by chi-square test. *p* < 0.05 was considered statistically significant.

## 3 Results

### 3.1 Baseline information of the patients

The baseline clinical characteristics of the US group and the MB group were summarized in Table 1. There were no significant differences in age, BMI, LOS, male-female ratio, the scope of surgery, the number of lesions, LNM and pathological pattern between the two groups of patients (*p* > 0.05).

**Table 1:** General clinical features.

Group	Age (y)	BMI^a^ (kg/m^2^)	LOS^b^ (d)	Gender (n)		Bilateral (n)		LNM^c^ (n)		Multiplicity(n)		Pathological pattern (n)		
				Male	Female	Yes	No	Yes	No	Yes	No	PTMC^d^	PTC^e^	FTC^f^
US^g^	36.38 ± 8	24.48 ± 3.17	7.51 ± 1.71	45	36	39	42	38	43	21	60	41	39	1
MB^h^	34.27 ± 8.45	25.00 ± 2.92	8.42 ± 2.66	48	25	30	43	31	42	15	58	22	46	5
*P* value	>0.05	>0.05	>0.05	>0.05		>0.05		>0.05		>0.05		>0.05		

^a^ Basic mass index.

^b^ Length of stay.

^c^ Lymph node metastasis.

^d^ Papillary thyroid microcarcinoma.

^e^ Papillary thyroid carcinoma.

^f^ Follicular thyroid carcinoma.

^g^ Ultrasonic scalper.

^h^ Maryland bipolar electrocoagulation.

### 3.2 Comparison of surgical conditions

The surgical conditions and postoperative complications were compared between the US group and the MB group, such as postoperative recurrent laryngeal nerve injury, intraoperative and postoperative bleeding, sympathetic nerve injury, hoarseness and cough in Table 2. There was no significant difference in the occurrence of postoperative complications between the two groups (*p* > 0.05). Based on the extent of resection during the surgery, we further compared the operation time and postoperative drainage volume of the patients in the two groups. Regardless of unilateral or bilateral, the operation time of the MB group was significantly shorter than that of the US group (*p* < 0.05), and the average postoperative drainage volume of the MB group was significantly higher than that of the US group (*p* < 0.05).

**Table 2:** Operation and postoperative complications.

Group	Recurrent laryngeal nerve signal (n)			Intraoperative bleeding (n)			Postoperative bleeding (n)		Hoarseness and coughing (n)		Horner syndrome (n)	
	Strong	Weak	No	No	Yes, and Transfer	Yes, but No Transfer	Yes	No	Yes	No	Yes	No
US	76	2	3	79	1	1	1	80	2	79	1	80
MB	71	2	0	73	0	0	0	73	1	72	0	73
*P* value	>0.05			>0.05			>0.05		>0.05		>0.05	

### 3.3 Comparison of parathyroid function

**Figure 3:**
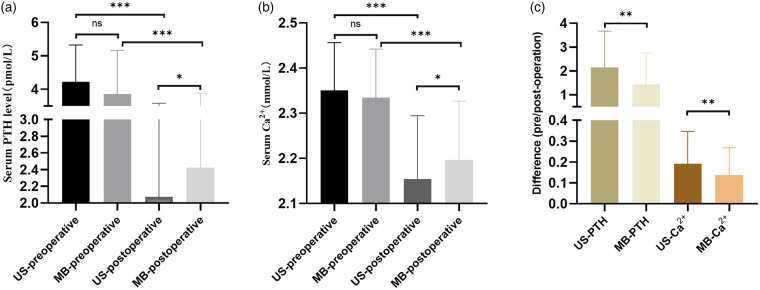
Compared with the function of parathyroid in US and MB. **(a)** The serum PTH level of US group and MB group before and after total thyroidectomy were compared, **(b)** The serum calcium level of US group and MB group before and after total thyroidectomy were compared, **(c)** The difference between PTH and serum calcium between US group and MB group before and after total thyroidectomy. *: *P* < 0.05, **: *P* < 0.01, ***: *P* < 0.001, ns: no statistical significance.

The parathyroid hormone (PTH) levels and blood calcium levels before and after the surgery were measured to evaluate the impact of the surgery on the patient's parathyroid function (Table 3). The patients were grouped according to the surgical scope, there was no significant difference in the levels of parathyroid hormone and blood calcium before unilateral lobectomy between the two groups (*p* > 0.05). The postoperative PTH level in the MB group was higher than that in the US group (3.24 ± 1.09 vs. 2.80 ± 1.51, *p* < 0.05), while there was no significant difference in the postoperative blood calcium level. Comparing the parathyroid function of patients who underwent total thyroidectomy in both groups revealed that the PTH levels after surgery in the US group and the MB group were (2.07 ± 1.51 vs. 2.42 ± 1.46, *p* < 0.05) (Figure 3a), and the blood calcium levels were (2.15 ± 0.14 vs. 2.20 ± 0.13, *p* < 0.05) (Figure 3b). The difference between preoperative and postoperative was significantly higher in the US group than in the MB group (*p* < 0.01) (Figure 3c).

**Table 3:** Comparison of PTH and blood calcium levels between the two groups ($\bar{x}\pm s$).

scope of surgery	Group	Operation time (min)	Volume of drainage (mL)	PTH (pmol/L)			Serum Ca^2+^ (mmol/L)		
				Pre-operation	Post-operation	Difference	Pre-operation	Post-operation	Difference
Total thyroidectomy	US (39)	252.18 ± 52.28	145.56 ± 45.64	4.23 ± 1.10	2.07 ± 1.51	2.15 ± 1.54	2.35 ± 0.11	2.15 ± 0.14	0.20 ± 0.15
	MB (30)	210.63 ± 61.92	178.30 ± 77.34	3.85 ± 1.31	2.42 ± 1.46	1.43 ± 1.32	2.33 ± 0.11	2.20 ± 0.13	0.14 ± 0.13
	*P* value	<0.05	<0.05	>0.05	<0.05	<0.01	>0.05	<0.05	<0.01
Unilateral lobectomy	US (42)	209.93 ± 45.77	136.57 ± 52.28	4.51 ± 1.57	2.80 ± 1.51	1.72 ± 1.32	2.38 ± 0.10	2.24 ± 0.16	0.16 ± 0.15
	MB (43)	146.60 ± 38.96	150.33 ± 73.80	4.21 ± 1.56	3.24 ± 1.09	0.97 ± 1.39	2.35 ± 0.18	2.27 ± 0.12	0.07 ± 0.20
	*P* value	<0.05	<0.05	>0.05	<0.05	<0.01	>0.05	>0.05	<0.01

## 4 Discussion

Surgery is the first choice for the treatment of PTC, and some patients need to be treated with radioiodine ablation (I-131) and thyroxine after surgery (18). The introduction of robotic surgical systems has provided more options for patients with early-stage thyroid cancer (19). The Da Vinci robotic surgical system integrates three-dimensional high-resolution imaging, a robotic arm with an endowrist rotation function, and an intuitive synchronous control system, allowing surgeons to perform more precise operations in narrow spaces (20). The most representative robotic thyroid surgery methods include bilateral axillary breast approach (BABA), transaxillary (TA), transoral robotic thyroidectomy (TORT) and retroauricular (RA) approach (21–24). Our team has carried out a total of 224 cases of robotic thyroidectomy since 2023, and has rich experience in robotic surgery based on membrane dissection, laying the foundation for exploring the application of bipolar electrocoagulation cutting technology in Da Vinci robotic thyroid cancer surgery. For more effective protection of the parathyroid gland, we used a “negative visualization” technique with nano-carbon tracer and real-time “positive visualization” with intravenous injection of indocyanine green during surgery. In addition, the RLN was monitored throughout the operation for better nerve protection. There was no significant difference in the general clinical characteristics between the two groups. Based on these findings, we further compared the efficacy and postoperative complications between US and MB groups.

US is the main dissection tool for minimally invasive surgery (25), and electrical energy is converted into mechanical energy and transmitted in the form of a sine wave that acts on the front of the metal cutter head, which produces a mechanical oscillation of 50∼100 μm in amplitude, resulting in frictional heat and shear force to both sides due to tissue tension (26). Bipolar electrocoagulation cutting technology is one of the most used surgical cutting hemostasis devices in clinical practice. The MB of Da Vinci robotic surgical system can be used for finer operations with the help of the mechanical arm with endowrist rotation function (27). Its energy radiation range is limited to the tiny area around the tip of the electric coagulation forceps, with little thermal damage to the surrounding tissue (28). In the MB group, the operation time significantly decreased, whereas the postoperative drainage volume increased. Compared with the US group, there was only 1 case of intraoperative tunnel bleeding in the MB group. Although there was no significant difference, MB was used to successfully stop bleeding during the operation, and there was no transformation to open or endoscopic surgery. We can conclude that MB cutting technology based on membrane dissection made the operation more flexible and faster due to the internal joint and finer tip, which effectively shortened the operation time. On the other hand, the MB technique leads to limited area of tissue coagulation necrosis due to the small radiation range, while increasing the possibility of tissue exudation, which is more obvious compared to the US group. The tissue is exposed more widely during the operation due to the enhanced visualization and refined anatomy. The fascia cavities established after fine capsule dissection using bipolar technology have more layers and can effectively discharge the exudate. Under the premise of improving the surgical precision, the increase in drainage volume did not affect the postoperative recovery and healing of the patients, and there was no significant difference in the length of hospital stay. Consistent with the above findings, the application of MB in robot-assisted gastrectomy and total mesenterectomy has achieved similar results. Moreover, its application in gastrectomy has been widely promoted due to its good surgical effect and low complication rate (29).

Impaired parathyroid function is the most common postoperative complication in thyroid cancer surgery (30), result in transient or permanent hypoparathyroidism in 30% of cases (31). The parathyroid gland is located between the true and false capsule of the thyroid gland, closely adjacent to the anatomical location of the thyroid gland, and its color is yellowish brown, which makes it difficult to distinguish from adipose tissue. Therefore, the protection of the parathyroid gland requires more fine dissection of the thyroid capsule. The US used by Da Vinci robotic surgical system lacks flexible internal joints, which cannot fully exploit the advantages of the robotic surgical system. Furthermore, the energy generated by US during the cutting process spreads within a range of approximately 1 cm tissues and produces different degrees of thermal effects (32). Thermal injury easily occurs during the surgery on the thyroid capsule, leading to parathyroid injury, RLN paralysis, and even permanent damage (33). MB just makes up for this lack. With the help of the high-definition magnified three-dimensional view and the robotic arm with degrees of freedom in robotic surgery, MB could be used to operate flexibly in a narrow space and completely filter the tremor, and it could complete the fine dissection of the capsule and the separation and suture in difficult angles (34). Thermal injury, destruction of the blood supply or accidental resection of the parathyroid gland during surgery can lead to functional impairment, which manifests as a decrease in serum PTH and serum calcium (35). In the research, we closely observed the function of parathyroid gland in the two groups before and after operation. Comparing the parathyroid function of patients who underwent total thyroidectomy in both groups revealed that the PTH levels and blood calcium levels in MB after surgery were higher than those in the US group. It could be concluded that the use of MB to separate and cut the thyroid caused less damage to the parathyroid gland. In recent years, many convenient operation methods have been tried based on fine capsule dissection to protect the parathyroid gland. Saline is injected into the subthyroid capsule at the level of the cricoid cartilage to enlarge the pore between the thyroid and parathyroid glands, and saline dilutes the yellow fat, allowing surgeons to better distinguish the parathyroid gland from adjacent tissues (36). Infrared autofluorescence has also assisted surgeons in realizing their potential for detecting and preserving parathyroid glands, significantly reducing the incidence of early postoperative hypocalcemia, but not improving postoperative low PTH levels (37). So far, the use of carbon nanoparticles and indocyanine green in thyroidectomy has gained wide consensus. Carbon nanoparticles were injected into the thyroid tissue 24 h before surgery to identify the parathyroid gland during central lymph node dissection (38), intraoperative intravenous injection of indocyanine green makes the blood vessels of parathyroid glands positively developed, which plays a crucial role in the visualization and identification of parathyroid glands (15). As a result, the use of MB to refine the dissection of the thyroid capsule based on the combination of nano-carbon and indocyanine green, can maximize the preservation of the inferior thyroid artery to the surface of the capsule, and further protect the parathyroid and blood supply. There are also disadvantages during MB manipulation, such as the ease of adhesion of the forceps head to tissue aggregation into eschar. Our experience on the use of bipolar electrocoagulation was to keep the two ends of the bipolar forceps as close as possible, which can reduce eschar formation and increase the coagulation effect. In addition, it is necessary to deal with the changes in the operation in real time. Considering that the space of visualization becomes smaller based on fine anatomy, the intensity of heat diffusion is enhanced, and the vigilance of blood vessels, nerves, and parathyroid gland injuries cannot be relaxed because of fine anatomy. While fully exerting its advantages in flexibility and exposure of the surgical field during the surgery, we also need to reduce the impact of the disadvantages. In the study, there was no sufficient reason to prove the neuroprotective effect of MB. We will continue to explore the clinical application value of Maryland bipolar coagulation forceps in the future.

## 5 Conclusion

In conclusion, Maryland bipolar coagulation in robotic thyroid cancer surgery could significantly shorten the operation time and significantly reduced the incidence of impaired parathyroid function after thyroidectomy. Therefore, MB cutting technique based on membrane dissection may be more suitable for robotic thyroid cancer surgery than ultrasonic scalpel cutting technique with fewer surgical complications.

## Data Availability

The raw data supporting the conclusions of this article will be made available by the authors, without undue reservation.
